# Exploring the Gut Microbiota: Key Insights Into Its Role in Obesity, Metabolic Syndrome, and Type 2 Diabetes

**DOI:** 10.1210/clinem/dgae499

**Published:** 2024-07-23

**Authors:** Sabitha Sasidharan Pillai, Charles A Gagnon, Christy Foster, Ambika P Ashraf

**Affiliations:** Center for Endocrinology, Diabetes and Metabolism, Children's Hospital Los Angeles, Los Angeles, CA 90027, USA; Department of Pediatrics, Keck School of Medicine, University of Southern California, Los Angeles, CA 90033, USA; University of Alabama at Birmingham Marnix E. Heersink School of Medicine, Birmingham, AL 35294, USA; Department of Pediatrics, The University of Alabama at Birmingham, Birmingham, AL 35294, USA; Department of Pediatrics, The University of Alabama at Birmingham, Birmingham, AL 35294, USA

**Keywords:** gut microbiota, gut microbiome, obesity, metabolic syndrome, type 2 diabetes

## Abstract

The gut microbiota (GM), comprising trillions of microorganisms in the gastrointestinal tract, is a key player in the development of obesity and related metabolic disorders, such as type 2 diabetes (T2D), metabolic syndrome (MS), and cardiovascular diseases. This mini-review delves into the intricate roles and mechanisms of the GM in these conditions, offering insights into potential therapeutic strategies targeting the microbiota. The review elucidates the diversity and development of the human GM, highlighting its pivotal functions in host physiology, including nutrient absorption, immune regulation, and energy metabolism. Studies show that GM dysbiosis is linked to increased energy extraction, altered metabolic pathways, and inflammation, contributing to obesity, MS, and T2D. The interplay between dietary habits and GM composition is explored, underscoring the influence of diet on microbial diversity and metabolic functions. Additionally, the review addresses the impact of common medications and therapeutic interventions like fecal microbiota transplantation on GM composition. The evidence so far advocates for further research to delineate the therapeutic potential of GM modulation in mitigating obesity and metabolic diseases, emphasizing the necessity of clinical trials to establish effective and sustainable treatment protocols.

## Introduction

The gut microbiota (GM), consisting of trillions of microorganisms in the gastrointestinal tract (GIT), significantly influences the development of obesity and related metabolic conditions such as type 2 diabetes (T2D), metabolic syndrome (MS), and metabolic dysfunction associated fatty liver disease (1-4). We aimed to provide a broad overview of the topic by conducting a thorough literature review through PubMed and Google Scholar, focusing on significant studies published up to 2005. The review examined the nature of the GM, factors that disrupt its balance (dysbiosis), and how such disturbances contribute to the onset of obesity, T2D, and MS.

### Human GM: Diversity, Development, and Impact on Health

The GM refers to the community of microorganisms residing in the GIT consisting of commensal, symbiotic, and pathogenic microorganisms while the microbiome encompasses all the genetic material of that microbiota (5, 6).

The human GM consists of approximately 100 trillion cells, which is 10 times the number of human cells (5, 7, 8). The density and composition of the GM increase from the upper to the lower intestines, with the highest diversity found in the colon (9). The composition of these microbial communities varies across different body sites and is influenced by various factors including host genetics, age, sex, weight, diet, immune system, oxygen levels, pH, bile acids, gastrointestinal transit time, mucus production, disease states, medications, probiotics, certain surgeries like gastric bypass, and environmental factors (9-15).

The colon, with its high transit time, favorable pH, low cell turnover, and redox potential, is particularly conducive to bacterial proliferation (16, 17). The healthy human GM comprises over 1000 bacterial species, predominantly from 6 phyla: Firmicutes, Bacteroidetes, Actinobacteria, Proteobacteria, Fusobacteria, and Verrucomicrobia (3, 18).

Colonization begins shortly after birth and progresses into adulthood. The GM composition varies in early life, influenced by delivery mode, feeding method, and diet with differences noted between vaginally born and cesarean-born infants. The maternal microbiome during pregnancy and whether the infant is breastfed or formula-fed also play crucial roles (13-15). By the age of 3, a more adult-like GM pattern is established (5). A diverse GM is rich in number and variety. The GM undergoes significant changes and transitions during childhood before reaching a relatively stable adult-like state (19-22).

A Japanese study investigated age-related changes in GM composition in 367 healthy individuals aged 0 to 104 years by analyzing the fecal samples. Significant GM transformations occurred during childhood (<20 years) as it matured and again after 70 years of age, shifting to an “elderly type.” Actinobacteria abundance markedly decreased after weaning and continued to decline with age. Firmicutes became dominant postweaning but were less abundant in children under 4 years compared to older subjects. Bacteroidetes and Proteobacteria increased after age 70 years, opposite to the Firmicutes trend. Distinct coabundance groups (CAGs) dominated at different life stages: Bacteroides, Eubacterium, and Clostridiaceae CAGs (elderly associated); Enterobacteriaceae CAGs (infant and elderly-associated); Bifidobacterium CAGs (infant/child-associated); Lachnospiraceae CAGs (adult-associated); and Megamonas and Peptoniphilus CAGs (relatively enhanced in the elderly). Sequential changes occurred in Bacteroides, Lachnospiraceae, and Bifidobacterium CAGs in the GM during childhood and adolescence (23). These changes likely reflect the interplay between the developing GM and host physiological factors across the lifespan.

A study from the Netherlands compared the GM composition and functional potential between children aged 9 to 12 years (the Generation R Study) and adults aged 46 to 88 years (the Rotterdam Study). Children exhibited lower GM diversity and higher levels of genus *Bacteroides*, while adults had an increased abundance of genus *Blautia*. Children's GM showed overrepresentation of pathways related to glycan degradation and riboflavin, pyridoxine, and folate biosynthesis. Adults’ GM had higher abundances of pathways involved in carbohydrate metabolism, beta-lactam resistance and thiamine and pantothenate biosynthesis. This suggests a shift from predominantly catabolic pathways in children to more biosynthetic pathways in adults (21).

The GM in healthy adults tends to be stable, shaped more by environmental factors than by host genetics (14). GM disruptions due to external factors can alter the symbiotic relationship with the host, potentially leading to the development of metabolic diseases (24).

### GM and Host Physiology

Large microbiome projects, such as the Human Microbiome Project in the United States and the Metagenomics of the Human Intestinal Tract in Europe, which utilized advanced analytic methods, such as high-throughput sequencing and metagenomic data analysis, have significantly contributed to our understanding of the human microbiota's organismal and functional data (3, 25).

### GM, Diet, and Metabolic Pathways

**Figure 1.**
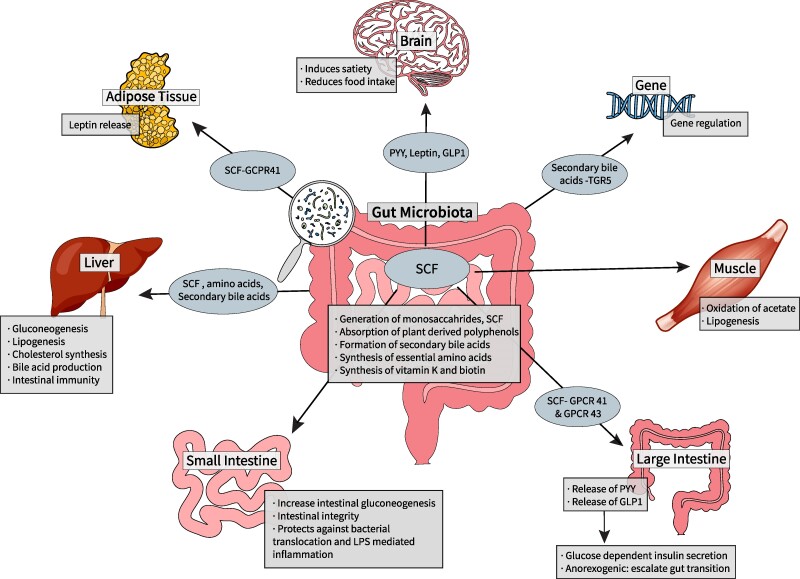
Gut microbiota and host metabolism: the gut microbiome plays a crucial role in the metabolism of dietary components. Undigested carbohydrates, proteins, and fats from the diet reach the colon and serve as substrates for bacterial fermentation. Gut bacteria possess a diverse array of enzymes that break down complex dietary components into metabolites like SCF, gases, and other bioactive compounds. These microbial metabolites can influence host energy balance, appetite regulation, and metabolic processes. For instance, SCF produced from fiber fermentation can modulate host energy harvest, satiety hormones, and glucose homeostasis. Additionally, the gut microbiome is involved in the metabolism of polyphenols, bile acids, and vitamins, further impacting host metabolism and health. Abbreviations: GLP-1, glucagon-like peptide 1; GPCR, G protein coupled receptor; PYY, peptide YY; SCF, short-chain fatty acid; TGR-5, Takeda growth factor receptor 5.

The GM plays a crucial role in energy extraction from the diet and synthesizes metabolites that function as signaling molecules, which regulate the neuro-immune-inflammatory axes, linking the gut to other organ systems (Fig. 1) (3, 22). Humans lack the enzymes needed to digest complex carbohydrates and depend on GM to digest complex dietary components like dietary fiber and starch, generating monosaccharides and short-chain fatty acids (SCFs) through hydrolysis and fermentation of undigested food components. SCFs, including butyrate, propionate, and acetate, provide about 10% of the human body's caloric needs and 70% of the adenosine triphosphate produced in the colon (3, 5, 17, 22, 25, 26).

Acetate, in particular, is utilized by muscle and adipose tissues, while butyrate supports the intestinal epithelial cells and maintains intestinal integrity by promoting tight junction proteins and mucin production, thereby preventing harmful bacterial translocation and lipopolysaccharide (LPS)-mediated inflammation (17, 18, 27). Butyrate and propionate promote intestinal gluconeogenesis (1, 27) protecting against diabetes and obesity by positively regulating glucometabolic pathways and hepatic gluconeogenesis (27, 28). SCFs also regulate the expression and action of anorexigenic hormones like glucagon-like peptide-1 (GLP-1) and peptide YY (PYY) through activation of G protein coupled receptors (GPCRs). GPCR41 activation releases PYY and leptin, while GPCR43 activation releases GLP-1 and GLP-2, stimulating glucose-dependent insulin secretion, increasing gut transit time and satiety, and reducing food intake (18, 22, 28).

Microbial lipases degrade triglycerides and phospholipids into their polar head groups and free lipids (17). Unmetabolized SCFs reach the liver as precursors for gluconeogenesis (propionate) and lipogenesis and cholesterol synthesis (acetate and butyrate) (17). High levels of SCFs inhibit lipoprotein lipase (LPL) in the small intestine, reducing fatty acid release and promoting triglyceride uptake, leading to increased fat storage (22). Acetate and butyrate promote fatty acid oxidation and energy expenditure through their activation of 5′ AMP-activated protein kinase (18).

The GM metabolizes bile acids, converting unabsorbed primary bile acids to secondary bile acids via colonic microbiota's bile salt hydrolases (3), which enhance local intestinal immunity by inhibiting pathogenic growth (17).

The GM regulates the endocannabinoid system, affecting host metabolism, energy, immunity, inflammation, and glucose and lipid metabolism. Conversely, the host's endocannabinoid system influences GM composition and activity, controlling energy expenditure and food intake via the gut-brain axis (29, 30).

Dietary amino acids are absorbed by intestinal epithelial cells and the bacterial lumen for survival, bacterial constituent synthesis, and metabolism. Undigested proteins in the small intestine undergo fermentation in the colon by the GM. Additionally, the GM produces essential amino acids and vitamins like K and biotin for the host (3, 8).

The fermentation of dietary proteins and the resulting metabolites are influenced by the protein source (animal/plant), amino acid composition, gastrointestinal transit time, overall macronutrient ratios, and GM metabolism. Dietary proteins impact the GM by modulating amino acid metabolism, amino acid transport gene expression, and immune system regulation through interactions with toll-like receptors (TLRs), autoinducer-2, and nucleotide oligomerization domain-like receptors. These interactions affect the GM immune axis via various signaling pathways crucial for maintaining intestinal mucosal immunity and microbiota stability (8, 31). Imbalances in the aforementioned factors lead to the production of potentially harmful metabolites from protein fermentation and can produce harmful metabolites like indoles, phenols, polyamines, hydrogen sulfide, amines, carnitine, and ammonia (32).

GM metabolites and proteins significantly influence host metabolism. Indole-propionic acid enhances insulin secretion and sensitivity, reducing the risk of T2D. Another metabolite N-acyl amide, mimics endocannabinoids, influencing glucose metabolism via GPCR 119 binding. GM-secreted proteins also regulate host function, such as ClpB from *Escherichia coli*, which mimics the host α-melanocyte-stimulating hormone to regulate appetite. In humans, Amuc_1100, from *Akkermansia muciniphila* (*A. muciniphila*) enhances gut barrier function with increased goblet cell density through TLR2. GM-synthesized neurotransmitters such as catecholamine, histamine, γ-aminobutyric acid, and serotonin or gaseous neurotransmitters such as nitric oxide and hydrogen sulfide also affect host metabolism (33).

### GM and Obesity and MS

**Figure 2.**
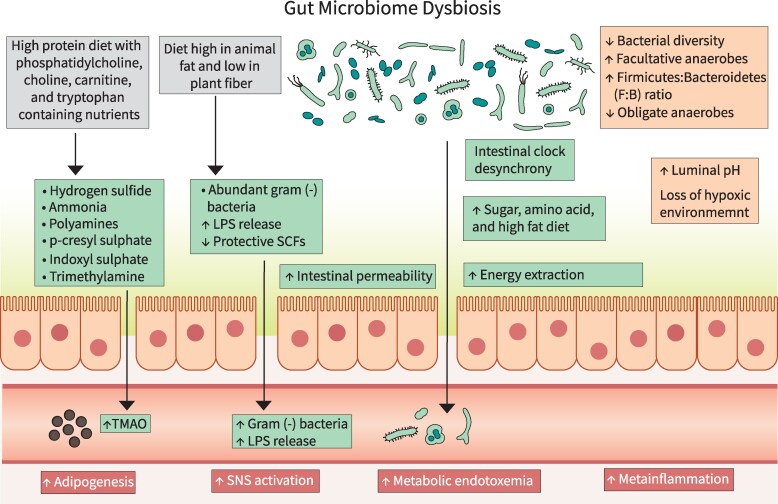
Mechanisms linking GM dysbiosis to obesity and metabolic syndrome: GM dysbiosis contributes to the development and progression of obesity and related metabolic abnormalities by influencing energy extraction from the diet, intestinal permeability, gut inflammation, proinflammatory markers, insulin sensitivity, circadian rhythm, sympathetic nervous system, and immune system. Abbreviations: GM, gut microbiota; LPS, lipopolysaccharide; SCF, short-chain fatty acid; SNS, sympathetic nervous system; TMA, trimethylamine; TMAO, trimethylamine oxide.

Recent literature suggests that GM dysbiosis could lead to many diseases including obesity, MS, and T2D. This occurs by decreasing microbial diversity and number, modulating paracrine and endocrine functions, changing the energy metabolism, influencing the satiety centers in the brain and pathways of inflammation, and altering the key functions performed by the microbes (34, 35) (Fig. 2).

The GM is considered a key regulator of host metabolism through the crosstalk between GM metabolites and host gene expression (27, 36, 37). Increased intake of high-energy, low-nutrient foods (high in sugar and fat) and decreased physical activity affect the GM (18, 34, 37). Interventional studies of weight reduction programs have observed an association between the GM and obesity. Weight loss following bariatric surgery or a dietary-exercise combined intervention improved obesity-associated GM-dysbiosis in study participants (34, 35, 38-43).

#### Enhanced Energy Extraction

The microbial composition affects the extraction and storage of energy from the diet, with certain bacteria being more efficient at harvesting energy, leading to weight gain (39). They regulate the production of SCF, modulate bile acid metabolism, and affect endocrine pathways.

Gram-positive Firmicutes were proposed to extract calories more efficiently from carbohydrates compared to gram-negative Bacteroidetes. Firmicutes increased the fermentation of otherwise indigestible carbohydrates into SCFs, which were then absorbed and utilized for gluconeogenesis and lipogenesis (39). The increased Firmicutes/Bacteroidetes (F/B) ratio has been extensively studied as a potential biomarker for obesity and metabolic diseases, but the findings remain controversial. Some studies have reported an increased F/B ratio in obese individuals compared to lean individuals (24, 44-46). However, other studies have failed to confirm this association or have even reported the opposite, with higher levels of Bacteroidetes and lower F/B ratios in obese subjects. Two meta-analyses concluded that the F/B ratio is not a consistent feature distinguishing lean from obese human GM (44). The relationship between the F/B ratio and these conditions is complex and not universally applicable (29, 47). Perhaps what matters is not just the fractionation but also the total bacterial count and the specific composition of the GM.

Human and mouse studies observed adverse effects on health with both low and high SCF concentrations, although the ideal SCF level in the body remains unclear. High SCF levels have been linked to obesity and insulin resistance (IR) in some studies, while others have reported that SCFs can improve insulin sensitivity and promote weight loss (48). Increased SCFs have been noted in fecal sampling of individuals with obesity compared to those without obesity (49). Increased SCF production in obese phenotypes depends on several factors, including substrate availability, gastrointestinal transit, mucosal absorption, gut health, GM production, and symbiotic relationships among different microbial groups (29, 49).

A particular microbial signature connected with a diagnosis of obesity has still not been recognized. The most common GM composition finding is reduction in abundances of butyrate-producing microbes and microbes associated with lower luminal LPS, improved intestinal barrier function, and reduced visceral fat mass (families *Rikenellaceae* and *Christensenellaceae*; genera *Bifidobacterium*, *Oscillospira*, and *Akkermansia*; and species *Alistipes finegoldii*, *Alistipes senegalensis*, and *Faecali prausnitzii*) along with increases in opportunistic pathogens such as hydrogen-producing bacteria and LPS-providing bacteria (families *Prevotellaceae*, *Coriobacteriaceae*, *Erysipelotrichaceae*, and *Alcaligenaceae*; genera *Roseburia*, *Fusobacterium*, *Escherichia-Shigella*, *Pseudomonas*, and *Campylobacter*; and species *Eubacterium dolichum*, Staphylococcus aureus) (29, 47).

Many studies propose that GM composition is related to weight, but certain dietary interventions appear to regulate more frequently some phyla, and, more notably, species in obesity GM composition, both at baseline and after interventions, correlated with clinical and metabolic features of the host (15). Genera *Lachnospira* and *Oscillospira* were negatively and *Faecalibacterium*, *Clostridiales*, and *Clostridium* were positively associated with weight. Multiple studies indicate that the human gut symbiont *Bacteroides thetaiotaomicron* can play a detrimental role in promoting obesity, impaired glucose metabolism, and metabolic disorders when present in higher abundance by promoting lipid digestion, absorption, and deposition through modulation of lipid metabolism pathways (50, 51).

Bacteroides species and chiefly *Prevotella* species belonging to Bacteroidetes phylum were associated with reductions in body weight, body mass index, waist circumference, fat mass percentage, and trunk fat percentage in various dietary models, including fiber-rich or high carbohydrate/high glycemic index diets. Subjects with an exorbitant abundance of *Bacteroides* were noted to have lower fiber-degrading potential than the *Prevotella* abundant subjects. *Prevotella* has been linked to both improved glucose metabolism with a high-fiber diet and IR with a high-fat diet through branched-chain amino acids (BCAA) production. However, conflicting associations exist between *Prevotella* abundance and obesity/metabolic diseases across different populations, with higher levels correlating with higher body mass index in Black and Hispanic individuals, particularly while on low-fiber diets. Further research is needed to understand these complex relationships and interactions with diet and host factors. *Akkermansia* is also associated with weight loss and positive effect on metabolic profile. Certain species like *Bacteroides eggerthii*, *A. muciniphila*, *Turicibacter,* and *Christensenella* were prevalent among individuals who successfully lost weight through commercial weight loss programs (15, 52, 53).

*Prevotella*, *Roseburia*, *Rumnicoccus*, and *Eubacterium rectale*, belonging to the phylum Firmicutes, and *Parabacteroides distasonis*, belonging to Bacteroidetes, are known to ferment complex polysaccharides to butyrate or succinate and acetate and have exhibited decreased levels in low-carb diets reliantly by the total amount of carbohydrate in cross-sectional studies. All these species appear to be a master signature of healthy diets, even though their abundance could be regulated by the preexistent GM as well as mechanisms of resilience to go back to an obesity microbiota phenotype (15).

Diet affects GM bidirectionally. Studies indicate that reduced *Prevotella copri* (*P. copri*) levels are linked to improved IR in those on the Mediterranean diet, whereas its abundance is associated with weight loss. While a Western diet reduces *P. copri*, it seems to provide metabolic benefits under a fiber-rich diet but not under a high-fat diet. This highlights how the type and source of macronutrients shape microbiota-disease connections (54, 55). Individuals with more severe metabolic phenotypes often have a marked reduction in genera with saccharolytic activity, leading to energy production from amino acids and the generation of BCAA, which impair glucose tolerance (15).

The GM enhances nutrient absorption by increasing the density of small intestinal villi capillaries and using microbial enzymes such as glycoside hydrolase, polysaccharide lyase, and carbohydrate esterase to boost intestinal monosaccharides uptake. The GM promotes lipogenesis by activating transcription factors like sterol regulatory element binding protein-1 and carbohydrate response element binding protein, which regulate lipid metabolism genes (39).

Peroxisome proliferator-activated receptor regulates the expression of fasting-induced adipocyte factor, a circulating LPL inhibitor synthesized by the intestinal epithelial cells and liver. The GM can hamper the fasting-induced adipocyte factor expression resulting in increased LPL activity and fat deposition in peripheral organs, contributing to obesity (49).

An overview of the systematic reviews on observational and interventional studies studying the microbiome's role in metabolic disorders such as T2D and MS observed less bacterial diversity in those with obesity vs those without obesity. Discerning bacteria coincided between metabolic disorders, with those showing overabundance often being involved in inflammation such as *Staphylococcus aureus*, *Ruminococcus,* and *Fusobacterium* (56). However, it is unclear whether GM changes cause obesity or if certain diets predispose individuals to develop a GM that promotes obesity.

#### Disruption of Circadian Rhythm

The circadian system regulates rhythmic behavior and synchronizes peripheral clocks in organs such as the GIT. In animal studies, specific genes like *Bmal1* control circadian rhythms and regulate various physiological aspects. Disruptions in these rhythms can alter GM rhythmicity and microbial metabolites, leading to metabolic issues like obesity, IR, and dyslipidemia (57).

Mice with *Bmal1* deficiency, and those subjected to simulated shift work, experienced desynchronization in peripheral clocks within the GIT, altered microbial rhythms, and metabolic changes affecting weight (57). Additionally, transferring fecal matter from jet-lagged humans to germ-free mice led to obesity and IR in the mice (39). Chronic high-fat diet consumption or antibiotic use also disrupts GM and leads to similar circadian disturbances and metabolic problems (58, 59).

#### Sympathetic Nervous System Stimulation

GM-derived metabolite SCFs (propionate) contribute to energy balance by causing sympathetic nervous system activation mediated through GPCR41 on the sympathetic ganglion (1).

#### Immune System

The GM plays a crucial role in local and systemic immunity, balancing immune responses and defending against pathogens. It locally modulates TLR expression, interacts with antigen-presenting cells, and influences T cell differentiation to maintain intestinal barrier integrity and systemic immune function. Systemically, the GM impacts immunity by influencing splenic CD4+ T cells and modulating antibody expression. GM-derived butyrate stimulates the production of anti-inflammatory cytokines [TGF-β, interleukin (IL)-10, and IL-18] and promotes the differentiation of naïve T cells into regulatory T cells (17, 22, 26, 60). SCFs bind to gut epithelial receptors like GPCR41 and GPCR43, inhibiting the nuclear factor κB pathway and reducing the expression of proinflammatory cytokines (tumor necrosis factor-α, IL-6, and IL12) (11, 18, 22, 60).

GM aids in nutrient absorption by promoting small intestinal angiogenesis (22, 26) and helps absorb plant-derived polyphenols with antioxidant, anticancer, and/or anti-inflammatory properties through biotransformation. Other GM functions include gene regulation, xenobiotic metabolism, and behavior development (5, 17, 22).

The immature immune system in early life may exert different selective pressures on the microbiota compared to adults, leading to distinct microbial compositions. Studies done on animals suggest that malfunction of the innate immune system can result in altered GM leading to MS (61-63).

#### Inflammation of the Gut

Prior studies suggest a pivotal role of gut dysbiosis in the generation of chronic low-grade inflammation, also referred as meta-inflammation, which has been proposed as a major factor in the development of obesity and associated complications such as MS and T2D (1, 18, 64, 65). This meta-inflammation results in macrophage infiltration in metabolic tissues, mainly the liver and adipose tissue, which in turn stimulates the release of proinflammatory cytokines including tumor necrosis factor-α, IL-1, IL-6, and other chemokines from adipocytes (1). These proinflammatory cytokines through paracrine and/or autocrine action cause more cytokines and chemokines to release from adipose tissue and also promote lipogenesis (18).

A study from Mexico found that children with MS and T2DM, compared to healthy controls, exhibited an increase in facultative anaerobes like enteric and lactic acid bacteria, along with a decrease in obligate anaerobes such as *Erysipelatoclostridium*, *Shaalia*, and *Actinomyces* genera, which may cause loss of gut's hypoxic environment, enhanced gut microbial nitrogen metabolism, and increased generation of pathogen-associated molecular patterns. These metabolic derangements may precipitate stimulation of proinflammatory activity and worsen intermediate metabolism in the host, potentially contributing to the development of the risk factors for MS and T2D such as IR, dyslipidemia, and central adiposity (66).

#### Metabolic Endotoxemia and Bacteremia

A high-fat diet alters the GM, increasing gram-negative bacteria and reducing SCFs. This imbalance compromises intestinal integrity, allowing the translocation of bacteria and their components like LPS, flagellins, and peptidoglycans into the systemic circulation (18, 67). Increased chylomicrons in the intestine due to a high-fat diet further facilitate LPS infiltration into the bloodstream (24). This leads to the chronic low-level systemic presence of bacteria and LPS, resulting in metabolic bacteremia and endotoxemia (1, 18). Low gut microbial diversity and a high level of proinflammatory markers and microbiota-derived LPS are often associated with obesity and MS (67). Once in the circulation, gram-negative bacteria and LPS bind to TLR4 and CD14 receptors on innate immune cells, triggering a strong immune response. However, studies indicate that in the absence of CD14 and TLR4, a high-fat diet induced metabolic bacteremia and endotoxemia do not elicit an immune response (18).

Further research, including both in vitro and in vivo studies involving mouse models and humans, demonstrated that LPS could promote the proliferation and differentiation of preadipocytes (67). LPS injections have also been shown to cause weight gain, IR, and an increase in proinflammatory markers in both mice and humans (56, 68-70). However, the immune response requires a higher level of circulating LPS than what is typically released by bacteria, and currently, no studies have proficiently measured the exact amount of LPS generated by bacteria (67).

#### Role of Bacterial Metabolites

Increasing research into bacterial metabolites has highlighted their potential as biomarkers for diagnosing and predicting metabolic disorders (67). Specifically, the GM metabolizes nutrients like choline, phosphatidylcholine, and carnitine (found in red meat, eggs, dairy products, and salt-water fish), producing trimethylamine, which is then converted into trimethylamine oxide (TMAO) in the liver. Elevated levels of TMAO are linked to cardiovascular diseases due to its effects on cholesterol metabolism, vascular inflammation, platelet aggregation, and thrombosis (67, 71, 72). Additionally, metabolites like indoxyl sulphate and p-cresyl sulfate, derived from the microbial metabolism of tryptophan (found in foods like oats, bananas, prunes, milk, tuna, cheese, bread, poultry, peanuts, and chocolate), and LPS produced by *Enterobacteriaceae* in response to a Western diet are implicated in the progression of kidney disorders, cardiometabolic diseases, and metabolic endotoxemia (67). These findings indicate the significant but complex role of gut microbial metabolites in health and disease, though the specific impacts of tryptophan metabolites on MS risk factors and complications remain controversial and require further investigation (67).

### GM in T2D

**Figure 3.**
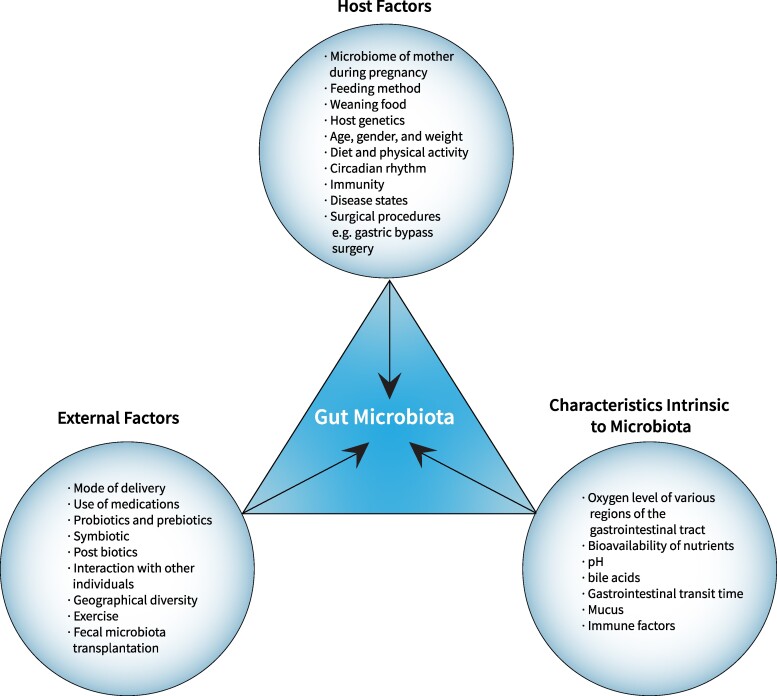
Factors influencing gut microbiota and potential therapeutic targets: this figure depicts the wide range of external and host factors and characteristics intrinsic to microbiota that affect the gut microbiota, as well as the potential areas for therapeutic targeting.

Dysbiosis in the GM has been linked to increased IR, a key feature of T2D. Figure 3 depicts the various external and host factors, as well as microbiota-intrinsic characteristics affecting the GM and potential therapeutic targeting.

Altered microbiota composition has been reported in subjects with T2D, including children (73, 66). A recent systematic review on GM in women with gestational diabetes mellitus (GDM) observed an association between GDM and GM dysbiosis, although no GDM-specific GDM-related GM was identified (74). A study on small intestinal microbiota infusions from lean donors to male recipients with MS showed increased insulin sensitivity and butyrate-producing GM levels in recipients after 6 weeks (75).

Meta-inflammation altered intestinal permeability with endotoxemia and reduced SCF production due to GM dysbiosis are associated with glucometabolic disturbances in subjects with T2D (64, 76). Defective lipoprotein metabolism in T2D also reduces LPS catabolism, increasing endotoxemia-related inflammation (24). GM dysbiosis may modify intestinal barrier functions and host metabolic pathways directly or indirectly related to IR in T2D (77). Patients with T2D exhibit decreased butyrate-producing GM compared to healthy controls (28), leading to impaired glucose tolerance and insulin sensitivity due to decreased GPCR release of PYY and GLP-1 (28).

Altered GM affects the metabolism of glucogenic amino acids, such as aromatic and BCAA promoting IR (78). Species like *P. copri* and *Bacteroides,* which promote BCAA biosynthesis, are abundant in T2D subjects (79). A study in Danish individuals demonstrated increased levels of BCAA in those with IR, linked to a GM with enhanced BCAA biosynthesis and lacking bacterial inward transporters for BCAAs (80). *P. copri* was identified as a key species driving the association between GM BCAA synthesis and IR, exacerbating glucose intolerance and reducing insulin sensitivity in mice (80).

In T2D subjects, there is a reduction in bacteria protecting the intestinal barrier, such as *A. muciniphila* and *Faecali prausnitzii*, and an increase in bacteria that damage the intestinal barrier, such as *Escherichia coli* (79). Gut microbial metabolites such as TMAO may contribute to IR and T2D (79, 81). A human trial showed that a low-energy diet decreased choline and L carnitine levels, precursors of TMAO, resulting in better glucose tolerance in adults with obesity and overweight (79, 82). Another study observed increased levels of imidazole propionate, a microbially synthesized histidine-derived metabolite, in subjects with T2D, impairing glucose tolerance in mice (83).

LPS impairs insulin sensitivity via activation of TLRs (69) and modulating nucleotide oligomerization domain-like receptors in macrophages and dendritic cells, triggering proinflammatory transcription factors such as nuclear factor κB and interferon regulatory factors, resulting in inflammasome component transcription. These events collectively influence glucose and lipid homeostasis (67). Higher LPS and LPS binding protein levels have been observed in patients with diabetes compared to healthy controls (84).

### GM and Dietary Interventions

Dietary habits significantly influence the GM composition and microbial metabolites that regulate host metabolism (67, 85). Dietary factors such as fat composition, fiber types and food additives, and environmental influences related to high-fat, high-sugar diets can reduce GM diversity and the overall number of microbes (27, 86). A Western diet, which is rich in fat and sugar but low in fiber, is associated with GM dysbiosis. In contrast, adherence to a Mediterranean diet increases SCF levels (67).

Short-term dietary changes can rapidly alter human GM. A study with 10 healthy volunteers aged 21 to 33, who alternated between animal-based and plant-based diets over 5 consecutive days, found that the animal-based diet phase led to an increase in bile-tolerant bacteria and a decrease in fiber-fermenting bacteria (87). Mice fed with high-fat diets exhibited characteristic microbial ecosystems: low Bacteriodes abundance, obesity, and MS with dietary saturated fatty acids; increased Bacteroidaceae with monounsaturated intake; and increased Biﬁdobacterium with ω-3 polyunsaturated fatty acids intake (88). Human studies demonstrated an increase in SCF-producing bacteria with ω-3 polyunsaturated fatty acids intake, whereas a protein-rich, low-carbohydrate diet resulted in GM dysbiosis and an increase in harmful metabolites like branched chain fatty acids, p-cresol sulfate, indoxyl sulfate, and TMAO (89, 90).

The CORDIOPREV study found that both the Mediterranean and low-fat diets could resolve dysbiosis in patients with obesity and/or MS (91). Rodents fed high-fiber diets were protected against diet-induced obesity and metabolic complications, likely due to the release of GLP-1 and GLP-2 by microbial fiber fermentation metabolites (92). In humans, resistant starch supplementation for 8 weeks led to weight loss and improved IR in overweight individuals by modifying the GM, altering bile acid profiles, and decreasing inflammation through intestinal barrier restoration and lipid absorption improvement (93). Supplementation of butyrate improved body mass index in children aged 5 to 17 years with obesity compared to those treated with placebo (94). However, large cohort studies indicate that GM stability may vary over time, and stratification among populations may not be as distinct (95).

### GM and Common Medications

Antibiotics are a major external disruptor of GM impacting both pathogenic and commensal gut bacteria, especially in children. Prenatal antibiotic exposure can adversely affect a child's GM (96, 97). Studies indicate that antibiotic use during early life significantly increases the risk of overweight and obesity. However, a large multicenter study involving approximately 39 000 children observed no significant increase in weight gain difference up to age 7 between those exposed to antibiotics in the first 6 months of life and those not exposed (96, 98, 99). A randomized double-blind placebo-controlled trial involving children aged 12 to 36 months showed that14 days of azithromycin reduced GM richness and diversity, but no differences in GM composition were observed between azithromycin and placebo groups 13 to 39 months posttreatment (100). Nonantibiotic drugs such as antineoplastic drugs and proton pump inhibitors also affect GM (99). Conversely, GLP-1 agonists have a positive influence on GM, with preclinical and clinical trials showing that these drugs promote weight loss by modulating GM, likely through decreasing gastric transit time and altering the local pH and nutrient availability (101).

### Restoration of Healthy GM

The potential ways to attain a healthy GM include adopting healthy dietary habits and using prebiotics, probiotics, fecal microbiota transplant (FMT) or microbiota-derived metabolites (73, 85, 102).

Prebiotics are nondigestible food ingredients, such as oligosaccharides, dietary fibers, and other nondigestible carbohydrates, which promote the growth of beneficial GM. They are considered a potential target for combating obesity (103). Probiotics, found in foods or supplements containing live microorganisms, include yogurt, cheese, fermented foods, and dietary supplements. Symbiotics, a combination of both probiotics and prebiotics, improves the survival and activity of beneficial GM (99, 104). Some probiotics can aid in the recovery of tight junctions between epithelial cells and modulate gut inflammation. Both prebiotics and probiotics influence lipid metabolism and the immune system and help manage gut inflammation. They are safe for children and offer a noninvasive method to complement dietary and lifestyle changes. Certain bacterial strains, *A. muciniphila*, have shown promise in improving metabolic syndrome and obesity (105, 106) However, currently, there is no incontrovertible proof to substantiate the proposed benefits of prebiotics, probiotics, and symbiotics on GM (99, 107, 108). Ongoing research, including clinical trials, is crucial to determine the most effective strains and doses of prebiotics and probiotics for treating pediatric obesity and MS. Over-the-counter probiotics are not yet recommended for these conditions.

FMT from a healthy donor is thought to restore a healthy GM in patients with GM dysbiosis (99). Current data on FMT for the management of obesity, MS, or T2D are limited, with few published cases involving small numbers of participants and short follow-up durations. Randomized controlled trials that evaluated FMT in people with obesity/MS comparing FMT from lean donors with controls observed conflicting results (109-114). With FMT in obese patients, donor colonization occurred in over 90% of recipients, but weight loss did not occur (115). Unlike the acute dysbiosis caused by *Clostridioides difficile,* which FMT can effectively treat, obesity represents a chronic state of dysbiosis, influenced by multiple factors, including dysregulation of host-microbe cross-talk. Therefore, treating dysbiosis alone may not be sufficient to improve metabolic parameters in obesity, which instead requires a multimodal treatment approach. Factors such as diet can impact the GM ratio and diversity, leading to inconsistent FMT results in treating obesity. Thus, FMT is unlikely to be a standalone therapy in treating obesity and metabolic disorders. Potential applications of FMT include combination with incretin therapy or other treatments for long-term GM modification to restore central and microbial disturbances leading to obesity (116).

The data on the GM is heterogeneous and not always reproducible; studies show varying compositions of Firmicutes and Bacteroidetes are likely due to the fact that GM is influenced by both intrinsic host factors and external factors (117, 118). Future prospective studies involving large homogenous populations are warranted to assess the ideal dose, route and frequency, safety, and long-term efficacy of FMT in patients with obesity, MS, or T2D.

## Conclusion

An altered GM composition in obesity, MS, and T2D is associated with increased energy extraction from the nondigestible dietary carbohydrates, enhanced gut permeability, and increased generation of proinflammatory metabolites, such as LPS, precipitating systemic inflammation and IR. GM manipulation to modify its influence on the development of obesity, MS, and T2D is considered a promising and novel treatment approach. GM alteration can be attained by dietary modification or administration of probiotics, prebiotics, symbiotics, and/or FMT targeting gut dysbiosis improvement in obesity and metabolic disorders. Future clinical trials are required to understand the optimal dose and frequency of these interventions and their long-term influence on host metabolism.

## Data Availability

This is a review of the literature, and no research data are reported. Further inquiries can be directed to the corresponding author.

## Abbreviations

A. muciniphila: Akkermansia muciniphila
BCAA: branched chain amino acids
CAG: coabundance groups
F/B: Firmicutes/Bacteroidetes
FMT: fecal microbiota transplant
GDM: gestational diabetes mellitus
GIT: gastrointestinal tract
GLP-1: glucagon-like peptide 1
GM: gut microbiota
GPCR: G protein coupled receptor
IL: interleukin
IR: insulin resistance
LPL: ipoprotein lipase
LPS: lipopolysaccharide
MS: metabolic syndrome
P. copri: Prevotella copri
PYY: peptide YY
SCF: short-chain fatty acid
TLR: toll-like receptor
T2D: type 2 diabetes
